# Development and validation of a deep learning-enhanced prediction model for the likelihood of pulmonary embolism

**DOI:** 10.3389/fmed.2025.1506363

**Published:** 2025-02-06

**Authors:** Yu Tian, Jingjie Liu, Shan Wu, Yucong Zheng, Rongye Han, Qianhui Bao, Lei Li, Tao Yang

**Affiliations:** ^1^Vascular Surgery Department, Shanxi Bethune Hospital, Shanxi Academy of Medical Sciences, Third Hospital of Shanxi Medical University, Tongji Shanxi Hospital, Taiyuan, China; ^2^School of Clinical Medicine, Tsinghua University, Beijing, China; ^3^Institute of Cardiovascular Diseases, The First Affiliated Hospital of Dalian Medical University, Dalian, China; ^4^Radiology Department, Shanxi Bethune Hospital, Shanxi Academy of Medical Sciences, Third Hospital of Shanxi Medical University, Tongji Shanxi Hospital, Taiyuan, China; ^5^Radiology Department, Tsinghua University Hospital, Tsinghua University, Beijing, China; ^6^Clinical Laboratory Department, Shanxi Bethune Hospital, Shanxi Academy of Medical Sciences, Third Hospital of Shanxi Medical University, Tongji Shanxi Hospital, Taiyuan, China; ^7^Vascular Department, Beijing Hua Xin Hospital (1st Hospital of Tsinghua University), Beijing, China

**Keywords:** pulmonary embolism, deep learning, deep venous thrombosis, risk assessments, clinical tool

## Abstract

**Background:**

Pulmonary embolism (PE) is a common and potentially fatal condition. Timely and accurate risk assessment in patients with acute deep vein thrombosis (DVT) is crucial. This study aims to develop a deep learning-based, precise, and efficient PE risk prediction model (PE-Mind) to overcome the limitations of current clinical tools and provide a more targeted risk evaluation solution.

**Methods:**

We analyzed clinical data from patients by first simplifying and organizing the collected features. From these, 37 key clinical features were selected based on their importance. These features were categorized and analyzed to identify potential relationships. Our prediction model uses a convolutional neural network (CNN), enhanced with three custom-designed modules for better performance. To validate its effectiveness, we compared this model with five commonly used prediction models.

**Results:**

PE-Mind demonstrated the highest accuracy and reliability, achieving 0.7826 accuracy and an area under the receiver operating characteristic curve of 0.8641 on the prospective test set, surpassing other models. Based on this, we have also developed a Web server, PulmoRiskAI, for real-time clinician operation.

**Conclusion:**

The PE-Mind model improves prediction accuracy and reliability for assessing PE risk in acute DVT patients. Its convolutional architecture and residual modules substantially enhance predictive performance.

## 1 Background

Pulmonary Embolism (PE) and Deep Venous Thrombosis (DVT) are the third most common acute cardiovascular diseases worldwide, following coronary heart disease and stroke (1). In epidemiological studies, the incidence of PE ranges from be 39–115 per 100,000 people annually (2). PE may cause symptoms such as shortness of breath, chest pain, syncope, and, in severe cases, sudden death. Its harmful effects primarily involve the impact on cardiopulmonary function. When the pulmonary artery is obstructed, the right ventricular load increases rapidly, leading to acute right ventricular dysfunction, cardiogenic shock, and even cardiac arrest.

Studies indicate that the all-cause mortality rate for PE is 1.9–2.9% at 7 days and 4.9–6.6% at 30 days (3). It is estimated that the mortality rate for untreated pulmonary embolism is about 30%, but with timely and effective treatment, the mortality rate can be reduced to less than 8%. Due to its insidious onset and often underestimated severity, PE is frequently unnoticed. Early, accurate diagnosis and prompt treatment are crucial for patients (4). The 2019 European Society of Cardiology Guidelines emphasize the importance of clinical evaluation for suspected PE and propose that assessing PE likelihood is critical for diagnosis (5). Research on predicting pulmonary embolism risk in DVT patients can help identify high-risk patient populations, optimize clinical treatment strategies, and reduce patient mortality and complications (6). Although the Wells score (7) and the revised Geneva score (8) are commonly used tools for assessing suspected PE, they do not specifically address the risk of PE development in patients with acute DVT. In clinical practice, accurately assessing the likelihood of PE in acute DVT patients is essential for understanding disease severity and progression. These assessments can guide clinicians in creating individualized management plans. Therefore, developing a dedicated PE risk prediction model for acute DVT patients is crucial.

In recent years, artificial intelligence (AI) has shown substantial potential in medical applications, particularly for disease risk prediction. Deep learning algorithms, such as convolutional neural networks (CNNs) and recurrent neural networks, are especially effective for processing medical imaging and time-series data (9–11). Furthermore, deep learning models can analyze electronic health record text data to identify high-risk factors in patients (12). However, challenges remain in applying AI to PE risk prediction, including data quality variability, model robustness, and interpretability (13). To address these issues, methods for optimizing feature selection and model architecture have been proposed, aiming to enhance interpretability without compromising accuracy (14).

This study aims to develop a novel deep learning model, PE-Mind, to more precisely evaluate PE risk in acute DVT patients. By incorporating multi-source clinical data, PE-Mind aspires to provide clinicians with a personalized decision-support tool. With further model validation, we expect PE-Mind to offer a viable solution in PE risk prediction, supporting improved clinical risk assessment.

## 2 Methods

**Figure 1:**
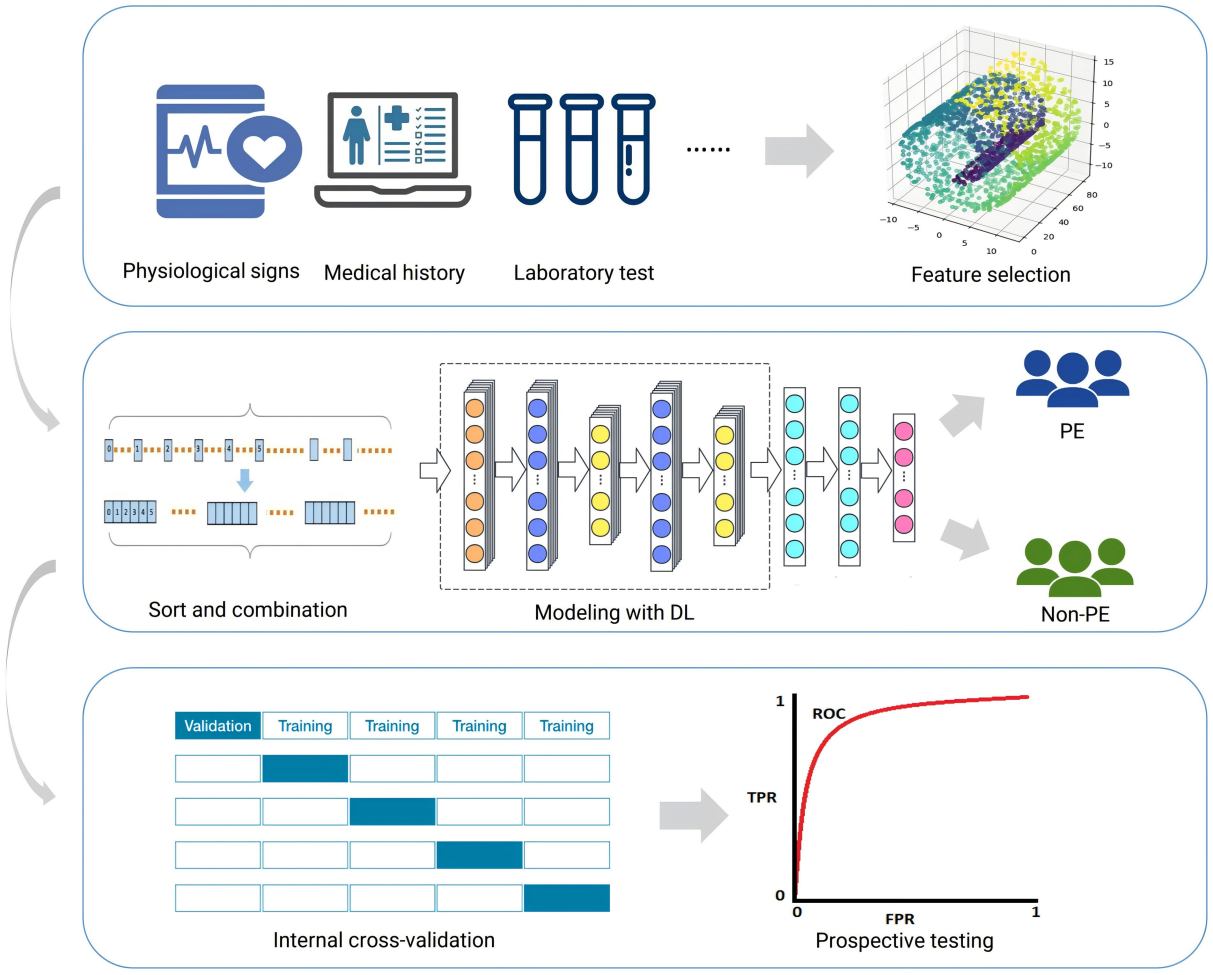
Workflow for this study. This figure outlines the sequential steps taken throughout the research, including data preprocessing, model construction, evaluation, and analysis. Each stage is highlighted to provide an overview of the study’s methodology.

The overall workflow of this study is shown in Figure 1.

### 2.1 Patients

This study was approved by the Ethics Committee of Shanxi Bethune Hospital (YXLL-223-097), and for all study participants the consent of the patients themselves or their guardians was obtained.

We collected data on 49 variables from patients diagnosed with acute lower extremity DVT in the Vascular Surgery Department of Shanxi Bethune Hospital from August 2020 to March 2023. Cases from August 2020 to December 2022 were retrospective data used for training the PE prediction model for DVT patients, and cases from January 2023 to March 2023 were prospective data used for testing the PE prediction model for DVT patients. The diagnostic criteria for acute lower limb DVT were onset time within 2 weeks and color Doppler ultrasound confirmation of the presence of a thrombus in the deep veins of the lower limbs. The diagnostic criteria for PE were computed tomography pulmonary angiography (CTPA) confirmation of the presence of a thrombus in the pulmonary artery. Based on these results, patients with PE were designated as the case group, and patients without PE were designated as the control group.

The inclusion criteria for this study were patients with acute lower limb DVT, and completion of CTPA examination within 3 days after DVT diagnosis. Exclusion criteria were patients who initially presented with PE, and the diagnosis of PE was made before the diagnosis of DVT, and special cases where CTPA examination could not clearly diagnose the presence of pulmonary embolism. Patients who refused to participate or had incomplete medical records or laboratory examination data were also excluded. Finally, 424 patients were included in the study, of which 379 were in the training set and 45 were in the validation set. The patient selection process is illustrated in Supplementary Figure 1.

### 2.2 Feature selection and processing

This study aims to develop a predictive model for PE risk by reducing the dimensionality of 49 clinical features associated with PE. Initially, we collected 49 clinical features from the clinical data of patients with acute DVT. To ensure equal contribution of each feature in the principal component analysis (PCA), we first standardized the data using the StandardScaler from sklearn.preprocessing (15–17).

Subsequently, PCA was applied to the standardized features, and the minimum number of principal components that explained at least 95% of the cumulative variance was selected. We further analyzed the contribution of each original feature to the principal components and excluded features with low variance (i.e., those with minimal variation in the patient population or limited contribution to PE risk prediction).

Ultimately, 37 features were retained, encompassing clinical, demographic, and laboratory variables, and their importance in the model was evaluated based on PCA loadings. These steps ensured that the selected features captured the primary variance in PE risk while minimizing redundancy. Through this dimensionality reduction approach, we effectively compressed the original 49 features, retaining the most predictive features, thereby enhancing the efficiency and accuracy of model training and prediction. Given that the selected clinical indicators were initially unordered, we transformed them into ordered data through grouping and sorting, in line with standard clinical data collection protocols, further enhancing the model’s predictive power.

### 2.3 Construction details of the predictive model

**Figure 2:**
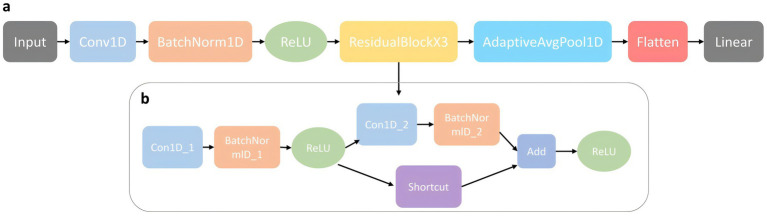
The model architecture of PE-Mind. **(A)** illustrates the overall network architecture, including input layers, feature extraction modules, and output layers. **(B)** provides a detailed view of the self-designed residual network architecture, showcasing its unique structural components and flow.

In this study, we propose a deep learning model called PE-Mind for predicting PE. The PE-Mind model utilizes a convolutional architecture, consisting of three custom-designed residual modules that effectively capture the intrinsic relationship between features and disease outcomes while preventing overfitting. The model’s input is a tensor of dimensions (batch_size, 1, 38), where batch_size represents the number of samples per batch, 1 represents the input channels, and 38 represents the input feature dimensions. The components of the PE-Mind model are as follows (network architecture illustrated in Figure 2):

1. **Convolutional Layer 1**: The first convolutional layer used 64 kernels of size 3, stride 1, and padding ‘same’, resulting in an output dimension of (batch_size, 64, 38).
2. **Batch Normalization Layer 1**: The output of Convolutional Layer 1 is batch-normalized to accelerate model convergence and prevent gradient explosion or vanishing.
3. **ReLU Activation Function 1**: The ReLU activation function is applied to the output of Batch Normalization Layer 1.
4. **Residual Modules**: Three residual modules comprise the core of the model. Each module includes two convolutional layers, two batch normalization layers, a shortcut connection, and a ReLU activation function. In the PE-Mind model, three residual modules are stacked sequentially to enhance the model’s expressiveness.
5. **Adaptive Average Pooling Layer**: An adaptive average pooling layer is applied after the final residual module to reduce the number of parameters and computational complexity, compressing the feature maps of each channel to 1 × 1 with an output dimension of (batch_size, 64, 1).
6. **Flatten Layer**: The output of the adaptive average pooling layer is flattened into a one-dimensional tensor with dimensions (batch_size, 64).
7. **Fully Connected Layer**: The fully connected layer transforms output from the flattened layer into final predictions, with two classes representing normal and PE states, yielding an output dimension of (batch_size, 2).

The PE-Mind model was trained using a cross-entropy loss function, which measures the difference between predicted and true probability distributions. The Adam optimizer was chosen to optimize model parameters, as it adaptively adjusts learning rates, accelerating convergence during training. Early stopping and learning rate decay strategies were utilized during training to prevent overfitting and accelerate convergence. A maximum of 200 training iterations was set, but in practice, models usually converge before reaching this maximum due to early stopping and learning rate decay strategies.

In each iteration, the training set was further subdivided into training and validation sets, and the Synthetic Minority Over-Sampling Technique(SMOTE)method balanced training data to address class imbalance. This approach ensured a nearly 1:1 ratio of normal to PE samples in the training set. During training, model parameters were updated using stochastic gradient descent with a batch size of 32. This approach balanced computation and convergence speeds while mitigating the influence of local minima. The initial learning rate was set to 1e-4, and learning rate adjustments were made dynamically based on validation set performance during training. Specifically, if validation performance did not improve after 10 consecutive iterations, the learning rate was halved. A maximum learning rate decay limit of five instances was set, with training terminating early once this limit was reached.

### 2.4 Construction of comparative models

In the study methods, we compared the designed model with other commonly used machine learning models for PE prediction. These models included LightGBM (LGBM), XGBoost (XGB), CatBoost, Random Forest (RF) and Multilayer Perceptron (MLP).

#### 2.4.1 LightGBM

LGBM is an efficient machine learning algorithm based on Gradient Boosted Decision Trees (GBDT) (18–20). In PE risk prediction, LGBM can handle numerous features and samples while maintaining high predictive accuracy and low computational complexity. Key parameters were set as follows: n_estimators = 400, max_depth = 5, and num_leaves = 32 to control the number of trees, depth, and leaf nodes.

#### 2.4.2 XGBoost

XGB is also a GBDT-based algorithm, which performs excellently in both competitions and practical applications (21, 22). In the field of PE risk prediction, XGB has been proven to effectively address imbalanced data problems and handle various types of features. The key parameters of XGB included n_estimators = 1,000, max_depth = 5, and learning_rate = 0.0001 to control the number of trees, depth, and learning rate.

#### 2.4.3 CatBoost

CatBoost is a novel GBDT-based algorithm specifically suited for datasets with categorical features (23). In PE risk prediction, CatBoost handles categorical features more effectively and overcomes the limitations of other GBDT algorithms in processing such features. In this experiment, key parameters were set as follows: iterations = 100, learning_rate = 0.001, and depth = 6 to control the number of trees, learning rate, and depth.

#### 2.4.4 Random forest

RF is an ensemble learning method that constructs multiple decision trees and aggregates their results for prediction (24). In PE risk prediction, RF effectively handle complex nonlinear relationships and demonstrate good robustness. In this study, we set n_estimators = 100, which determines the number of decision trees in the forest.

#### 2.4.5 Multilayer perceptron

Additionally, the MLP was also employed for comparison. This model is a supervised learning algorithm based on neural networks, suitable for handling nonlinear relationships (25). In PE risk prediction, the MLP can capture complex patterns hidden within the data, thereby improving predictive performance. Specifically, we chose two hidden layers, each containing 50 nodes, and set max_iter = 5,000 and learning_rate_init = 0.001 as key parameters.

### 2.5 Model evaluation

In the model evaluation phase of this study, a variety of metrics were used to comprehensively assess the performance of different models in PE risk prediction tasks. These metrics included accuracy (ACC), recall, precision (Prec), F1-score, and the area under the curve (AUC) of the receiver operating characteristic (ROC) curve. In this study, the classification threshold was set at 0.5. We conducted five-fold cross-validation on the training set data and calculated the mean values of each model for these metrics, accompanied by 95% confidence intervals (CI) to reflect the reliability of the model performances. Additionally, the ROC curves for each model were plotted to visually demonstrate the classification performance at different thresholds.

### 2.6 System development

Based on the best-performing PE risk prediction model, a practical web server named PulmoRiskAI was developed using the Streamlit 0.89.0 framework. A concise and intuitive user interface was first designed, allowing doctors and researchers to input patients’ clinical data easily. Users can easily input various indicators, such as age, gender, symptoms, signs, and relevant laboratory test results, through simple input boxes and dropdown menus. Basic validation and error prompts are provided to ensure the accuracy of the input data.

The operating environment for this study was the Windows 10 operating system, with hardware configurations including an NVIDIA RTX 3070 GPU (8GB VRAM), an AMD Ryzen 75800H processor (3.20 GHz), and Radeon Graphics. The programming language employed in the experiment was Python, based on the PyTorch 1.10.0 deep learning framework.

## 3 Results

### 3.1 Demographics and baseline indicators

The clinical data of 379 patients collected in this study served as the training set, while data from 45 patients were used as the test set. The dataset contained 49 baseline clinical features. Key features potentially related to PE risk were identified through detailed statistical analysis of these high-dimensional clinical features (see Supplementary Table 1 for baseline analysis results).

### 3.2 Results of feature screening and ranking combinations

Dimensionality reduction was performed on the 49 PE-related clinical features through PCA, ultimately screening out 37 principal components with a higher explained variance ratio. These principal components were related to various clinical features including surgical history, coagulation indicators (fibrinogen [FIB], prothrombin time [PT], and activated partial thromboplastin time [APTT]), history of coronary heart disease, history of hypertension, and their contributions are displayed in Supplementary Table 2. Analysis of each principal component revealed that surgical history had the highest absolute contribution (0.4102) in PC37, while other features such as FIB, PT, and APTT also had high contributions. This suggests that factors such as surgical history and coagulation indicators may play critical roles in predicting PE risk.

As described in the methods section, features were divided into six different categories, which allowed features with strong correlations to cluster together and facilitated PE-Mind’s capture of relationships among features. Within each group, features were ranked according to their importance. Subsequently, features from different subgroups were connected in the standard order of clinical information gathering (basic information, clinical manifestations and causes, personal medical history, family medical history, vital signs examination, laboratory examinations). This formed a structured feature sequence that helped the model better learn the relationships between features, thereby improving its predictive performance. The grouping and ranking results are shown in Table 1, with the corresponding baseline details provided in Supplementary Table 1.

**Table 1:** Grouped, sorted and concatenated clinical features for ordered input models.

Category	Feature	PCA Contribution
Basic information	Sex	0.132743
	Age	0.090734
Clinical manifestations and etiology	Presence of Etiological Factors	0.136228
	Unilateral Lower Limb Edema	0.051072
	DVT Location	0.044974
	Unilateral Lower Limb Pain	0.030719
	DVT Affected Side	0.025975
Personal medical history	Surgery History	0.410171
	Coronary Heart Disease History	0.240626
	Arterial Stenosis History	0.177364
	Arteriosclerosis History	0.141174
	Immobilization History	0.123982
	High Blood Pressure History	0.123497
	Venous Thrombosis History	0.109457
	Cancer History	0.088844
	Chronic Lung Disease History	0.074498
	Heart Failure History	0.052186
	Diabetes History	0.036445
	Cerebral Infarction History	0.029569
	Arterial Embolism History	0.029569
Family medical history	VTE Family History	0.189477
Vital signs	Hypertension History	0.217242
	Body Temperature	0.137686
	Blood Oxygen Saturation	0.10425
	Respiratory Rate	0.081267
	Heart Rate	0.066132
Laboratory tests	Fibrinogen	0.358052
	Prothrombin Time	0.348968
	Activated Partial Thromboplastin Time	0.310127
	D-Dimer	0.187352
	Anticoagulant Level III	0.170385
	Basophilic Count	0.152981
	Blood Platelet Count	0.131469
	Thrombin Time	0.125334
	Aspartate Aminotransferase	0.074987
	Serum Chlorine	0.073202
	Alanine Aminotransferase	0.072602

### 3.3 Results of 5-fold cross-validation for the prediction models

**Figure 3:**
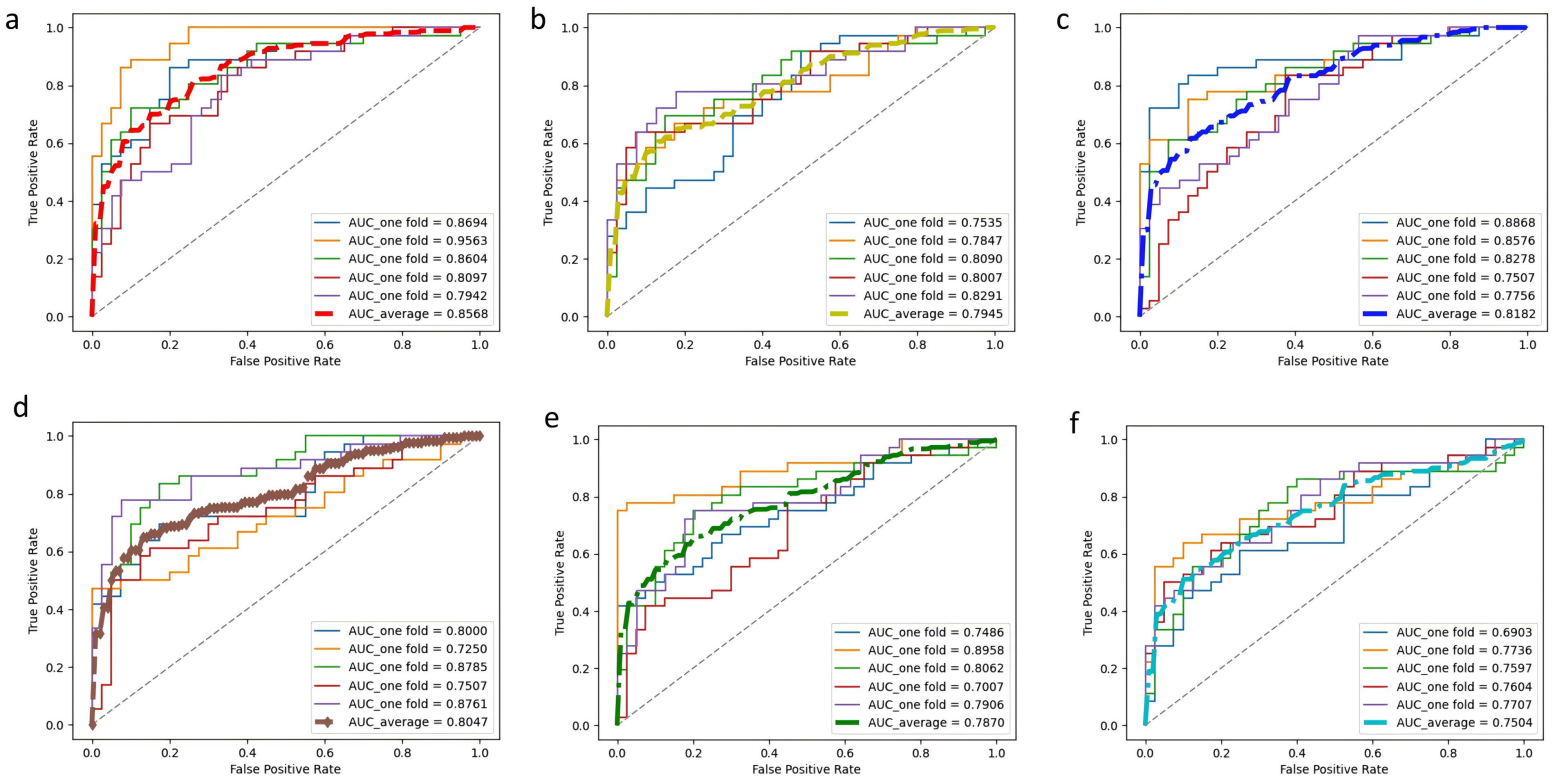
ROC curves and AUC values of each model in 5-fold cross-validation. Subplots **(A–F)** correspond to PE-Mind, LGBM, XGB, CatBoost, RF, and MLP, respectively, demonstrating each model’s performance in terms of sensitivity and specificity under cross-validation.

In this study, a deep learning model named PE-Mind, containing three custom-designed residual modules, was proposed to predict the risk of PE. To validate the performance of the PE-Mind model, it was compared with other popular machine learning models, including LGBM, XGB, CatBoost, RF, and MLP. These models were selected because they represent a broad range of machine learning and deep learning techniques commonly employed in clinical risk prediction tasks. MLP and Random Forest are traditional models that have been well-validated in medical contexts. LGBM and XGB are known for their strong performance in classification tasks, while catboost provides a state-of-the-art approach for complex feature learning. A 5-fold cross-validation was executed on the training set for all models, and the Acc, recall, Prec, F1 score, and AUC values were calculated for each result. The performance comparison of the models during cross-validation is shown in Table 2, and the corresponding ROC and AUC values are displayed in Figure 3.

**Table 2:** The results of 5-fold cross-validation for each model.

Model	Acc (%)	Recall (%)	Prec (%)	F1 (%)
PE-Mind	77.64 ± 4.19	79.44 ± 4.73	74.23 ± 3.67	77.22 ± 3.64
LGBM	73.37 ± 3.95	67.78 ± 3.45	74.52 ± 4.39	70.83 ± 3.87
XGB	73.34 ± 4.53	64.45 ± 4.62	76.75 ± 8.38	69.25 ± 5.99
CatBoost	74.96 ± 5.49	60.56 ± 6.83	82.31 ± 8.44	69.55 ± 7.15
RF	74.15 ± 7.76	62.22 ± 9.12	78.58 ± 8.79	69.23 ± 8.91
MLP	69.39 ± 3.58	57.22 ± 6.35	72.80 ± 4.15	63.84 ± 4.82

Each indicator is expressed as the mean ± 95% confidence interval (CI) of 5 experiments.

The results from the 5-fold cross-validation revealed that the PE-Mind model outperformed the other models in terms of Acc, recall, Prec, and F1 score. Specifically, the average Acc, recall, Prec, and F1 score of the PE-Mind model were 79.24, 79.45, 74.23, and 77.22%, respectively. In contrast, the performance indicators of the other models were lower. In terms of average AUC values, the PE-Mind model demonstrated a higher performance (0.8568), which was substantially higher than the other models, such as LGBM (0.7945), XGB (0.8182), CatBoost (0.8047), RF (0.7870), and MLP (0.7504).

Furthermore, the performance stability of the PE-Mind model was exceptional. In the five cross-validations, the variability of Acc, recall, Prec, and F1 score was small, indicating excellent robustness. For example, the range of accuracy was between 70.67 and 86.84%, and the range of recall was between 66.67 and 88.89%. This suggests that the PE-Mind model not only outperforms other models in overall performance but also maintains stable predictive performance across multiple runs. Conversely, other models, such as LGBM, XGB, CatBoost, RF, and MLP, exhibited significant fluctuations in performance across different validation sets. For instance, the Acc of the LGBM model ranged from 67.11 to 78.67%, while the recall of the RF model ranged from 44.44 to 75.00%. These results further emphasize the PE-Mind model’s robustness and stability.

### 3.4 Prediction models test results

**Figure 4:**
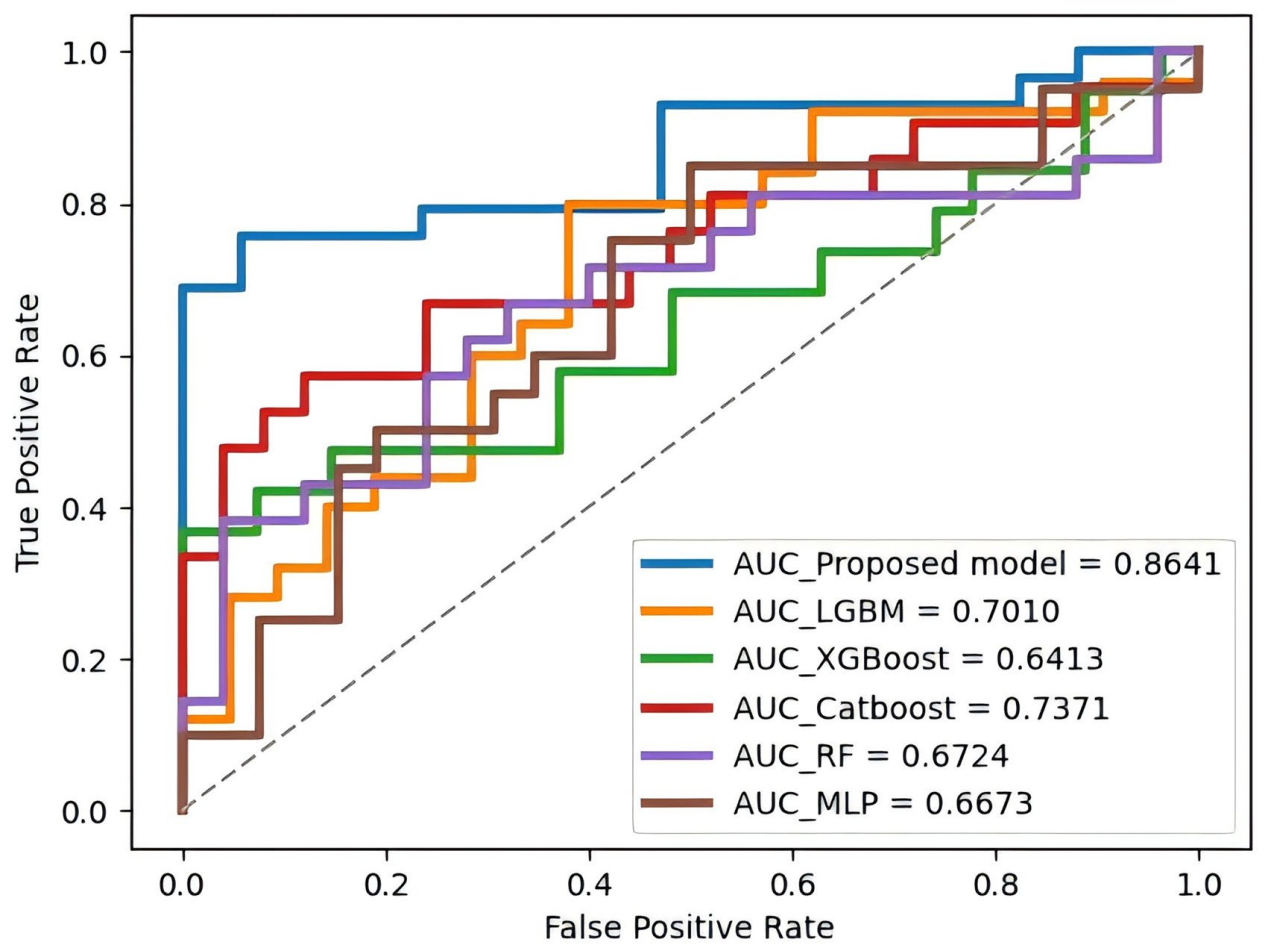
The ROC curve and AUC value of each model on the test set. This figure compares the predictive performance of all models when applied to unseen test data, highlighting their clinical applicability.

On the test set, the PE-Mind model exhibited outstanding generalization performance, significantly surpassing the other five machine learning models (detailed results are shown in Table 3, with corresponding ROC curves and AUC values in Figure 4). The test results showed that the PE-Mind model achieved the best performance in all evaluation metrics, with an Acc of 78.26% and an AUC of 0.8641, indicating its excellent generalization capability to accurately predict the risk of PE in new patient data. In comparison, the other five models demonstrated inferior performance. For example, the LGBM and XGB models showed lower Acc and AUC values, while the CatBoost and RF models had relatively lower recall and Prec. These results reflect that, compared to the PE-Mind model, other models may be inadequate in capturing key features and identifying underlying patterns when working with new patient data.

**Table 3:** The results of each model on the test set.

Model	Acc (%)	Recall (%)	Precision (%)	F1 score (%)
PE-Mind	78.26	68.97	95.24	80.00
LGBM	58.70	44.00	68.75	53.66
XGB	65.22	47.37	60.00	52.94
CatBoost	69.57	61.90	68.42	65.00
RF	65.22	61.90	61.90	61.90
MLP	60.87	60.00	54.55	57.14

Additionally, the PE-Mind model exhibited particularly remarkable performance in Prec, reaching 95.24%, which was considerably higher than other models. This suggests that the PE-Mind model is capable of substantially reducing the likelihood of false positives in identifying high-risk PE patients, thus avoiding unnecessary examinations and treatments. In contrast, the other five models may fail to adapt to the complexity and potential distribution differences of new patient data, resulting in inferior generalization performance. Therefore, the performance of the PE-Mind model on the prospective test set validated its superiority in predicting PE risk.

### 3.5 Development and demonstration of PulmoRiskAI

Building on the rigorous testing foundation of the model, a web server application called PulmoRiskAI was developed using the Streamlit framework. The application offers a clean and intuitive user interface, enabling clinicians and researchers to effortlessly input patient clinical data. Upon data entry, PulmoRiskAI automatically invokes the trained PE-Mind model to provide instant PE risk prediction for the patient.

To facilitate better understanding of the prediction results, a clear and intuitive visualization interface was designed (as seen in Supplementary Figure 2). Upon prediction completion, PulmoRiskAI presents the user with a risk probability prediction value, visually displaying the patient’s probability of developing PE. Moreover, a detailed risk assessment report is provided, including patient personal information, clinical features, and the predicted PE risk probability. These visualization tools aim to assist users in easily interpreting the prediction results, thereby offering improved diagnostic support. Furthermore, future efforts should focus on enhancing PulmoRiskAI’s features and iterating more powerful models to better serve patients and clinicians.

## 4 Discussion

This study developed a machine learning model, PE-Mind, aimed at accurately predicting the risk of PE, was undertaken. Leveraging the powerful computational capabilities of machine learning, we aimed to provide clinicians and researchers with a real-time and accurate tool to enhance diagnostic accuracy. To achieve this goal, a CNN was adopted as the fundamental architecture of the model, and three custom residual modules were designed to enhance the model’s expressive power. During model training and validation, five-fold cross-validation was performed, and performance metrics such as Acc, recall, Prec, F1 score, and AUC were calculated. The results demonstrated that the PE-Mind model outperformed other commonly used machine learning models. Furthermore, the PE-Mind model exhibited excellent generalization performance on the test set (AUC = 0.8641), indicating its potential for practical applications. Building upon the PE-Mind model, we further developed a practical web server called PulmoRiskAI. This interactive tool enables users to input patients’ clinical data for automated prediction of PE risk and provides intuitive visualization of the prediction results.

This paper conducted an investigation of 37 selected features related to PE. These features encompassed patients’ personal information, vital signs, medical history, laboratory tests, clinical manifestations, and etiological factors, providing abundant information sources for the PE-Mind model. Among these, surgical history emerged as one of the most important features, potentially closely associated with the occurrence of PE. This is also in line with the current mainstream prediction models for PE (7, 8). Surgery, as the most closely related factor, may be associated with the presence of Virchow’s triad - endothelial injury caused by surgical procedures, a hypercoagulable state during the perioperative period, and reduced blood flow due to prolonged bed rest after surgery. These three factors significantly increase the risk of venous thromboembolism (VTE), thereby increasing the risk of PE (26). Laboratory indicators, such as FIB, PT, and APTT, have a higher weight in the prediction model. These three coagulation test predictive values are very consistent with clinical features. In existing studies, it has been confirmed that abnormal FIB is strongly associated with the formation of venous thrombosis (27). A shortened APTT indicates a hypercoagulable state, which is an independent related factor for VTE (28). Including these three indicators in the model enhances the accuracy of predicting PE occurrence. Simultaneously, in the PE-Mind model, some vital signs, such as heart rate, blood pressure, and respiratory rate, may also be related to the occurrence of PE. These basic clinical indicators reflect the patient’s circulatory and respiratory functions and are also important indicators for monitoring PE patients in clinical practice. Furthermore, medical history features such as a history of coronary heart disease, hypertension, and diabetes may also be associated with risk of PE. These chronic diseases can contribute to vascular damage, inflammatory responses, and endothelial dysfunction, thereby increasing the likelihood of thrombus formation (29). Notably, a family history of VTE also held importance in the selection results, suggesting the potential role of genetic factors in the pathogenesis of PE. Through the analysis of these features, a better understanding of the mechanisms and potential risk factors underlying PE can be achieved.

A novel approach was employed to process the clinical feature data of patients, making it suitable for input into the PE-Mind model. Typically, 1D CNN is suitable for handling sequentially structured data, however the initially selected clinical features in this study were unrelated. Consequently, it was necessary to sequentially process the features to adapt them to this architecture. In this study, the top 37 clinically important features were grouped, sorted, and concatenated, successfully transforming unstructured data into logically structured data, thereby enhancing the model’s performance. This method aggregated features with strong correlations, aiding PE-Mind in better capturing the relationships among these features. Additionally, grouping facilitates the reduction of data redundancy and noise, enabling more effective learning of underlying data patterns. By sorting the features within each group according to their importance, the model focused on the most influential features for predicting PE, thereby improving accuracy. Subsequently, by sequentially concatenating the features within each group according to the clinical information gathering workflow, a structured feature sequence was formed, which enabled the model to better capture the correlations among the features. Normalizing the features before inputting them into PE-Mind helped eliminate data scale differences, and further improved model stability and performance, ensuring efficiency and accuracy in handling features of different scales.

The superior performance of the PE-Mind model in predicting PE risk can be attributed to its convolutional structure, custom-designed residual modules, the application of batch normalization and adaptive average pooling layers, and the effective training and evaluation strategies used. Firstly, 1D CNN demonstrated strong feature extraction capabilities in handling sequential data, which enabled more effective capturing of the relationships among features relevant to PE. Secondly, the custom-designed residual modules address the issue of vanishing gradients by introducing shortcut connections, thereby enhancing the model’s expressive power. Additionally, the application of batch normalization and adaptive average pooling layers ensured stability and computational efficiency during the training process. Simultaneously, effective training strategies, such as cross-entropy loss function, Adam optimizer, early stopping, and learning rate decay, were employed to achieve efficient convergence of the model and prevent overfitting. Finally, by applying the SMOTE technique, the ratio of normal to PE samples in the training set was balanced, enhancing the model’s generalization performance. These innovative aspects collectively contribute to the superior performance of the PE-Mind model in predicting the risk of PE compared to other mainstream models.

PulmoRiskAI holds significant promise in clinical application. By leveraging deep learning on a large amount of patients’ clinical feature data, it can accurately identify patients at risk, thereby enhancing diagnostic accuracy. This will help prevent incorrect and missed diagnoses, providing patients with more precise treatment plans. From a clinical practicality perspective, PulmoRiskAI assists clinicians in selecting more suitable treatment methods for patients. For instance, for patients identified to be at high risk of PE, clinicians can promptly take proactive treatment measures to reduce mortality. Conversely, for patients at low risk of PE, over-examination or overtreatment can be avoided. For example, low-risk patients could avoid CTPA scans, reducing unnecessary medical risks and resource consumption.

This study has several limitations. Firstly, the dataset used in this study was derived from a single center. Although prospective data were validated, this limits the assessment of the model’s generalizability across different regions and populations. Secondly, this study primarily focused on clinical features, Future research could incorporate additional factors, such as genetics and imaging to improve the predictive accuracy and applicability of the model. Furthermore, due to technical and data limitations, we were unable to fully explore the complex interactions among features, which may affect the predictive capabilities of the model. Future research could utilize more advanced deep learning techniques and larger, more comprehensive datasets to further optimize PulmoRiskAI.

## 5 Conclusion

This study developed PE-Mind, a deep learning-based predictive model for accurately assessing PE risk in patients with acute DVT. By integrating comprehensive clinical feature data, we transformed unstructured data into an ordered format suitable for model input. A time-based validation approach was applied to effectively evaluate the model’s generalizability, ensuring its reliability in practical applications. The PE-Mind model incorporates convolutional architectures and residual modules to enhance prediction accuracy. Based on this model, we have developed an online real-time platform, PulmoRiskAI, providing clinicians with a convenient, end-to-end risk assessment tool. Future work could explore the integration of additional data types, such as genetic information, and the application of more advanced deep learning techniques and larger datasets to further optimize PE-Mind’s potential for clinical application.

## Data Availability

The raw data supporting the conclusions of this article will be made available by the authors, without undue reservation.

## Supplementary material

SUPPLEMENTARY FIGURE 1 The flowchart of the patient selection process. This figure shows the criteria and steps for including or excluding patients, ensuring a clear understanding of the study cohort’s composition.

SUPPLEMENTARY FIGURE 2 Display of the operation interface of PulmoRiskAI. This figure provides a snapshot of the software interface, showcasing its user-friendly design and core functionalities for clinical application.
