# Supplementary figures and images for: Development and validation of a deep learning-enhanced prediction model for the likelihood of pulmonary embolism
Source: Front Med (Lausanne). 2025 Feb 6;12:1506363. doi: 10.3389/fmed.2025.1506363 (PMC11839595; doi:10.3389/fmed.2025.1506363)

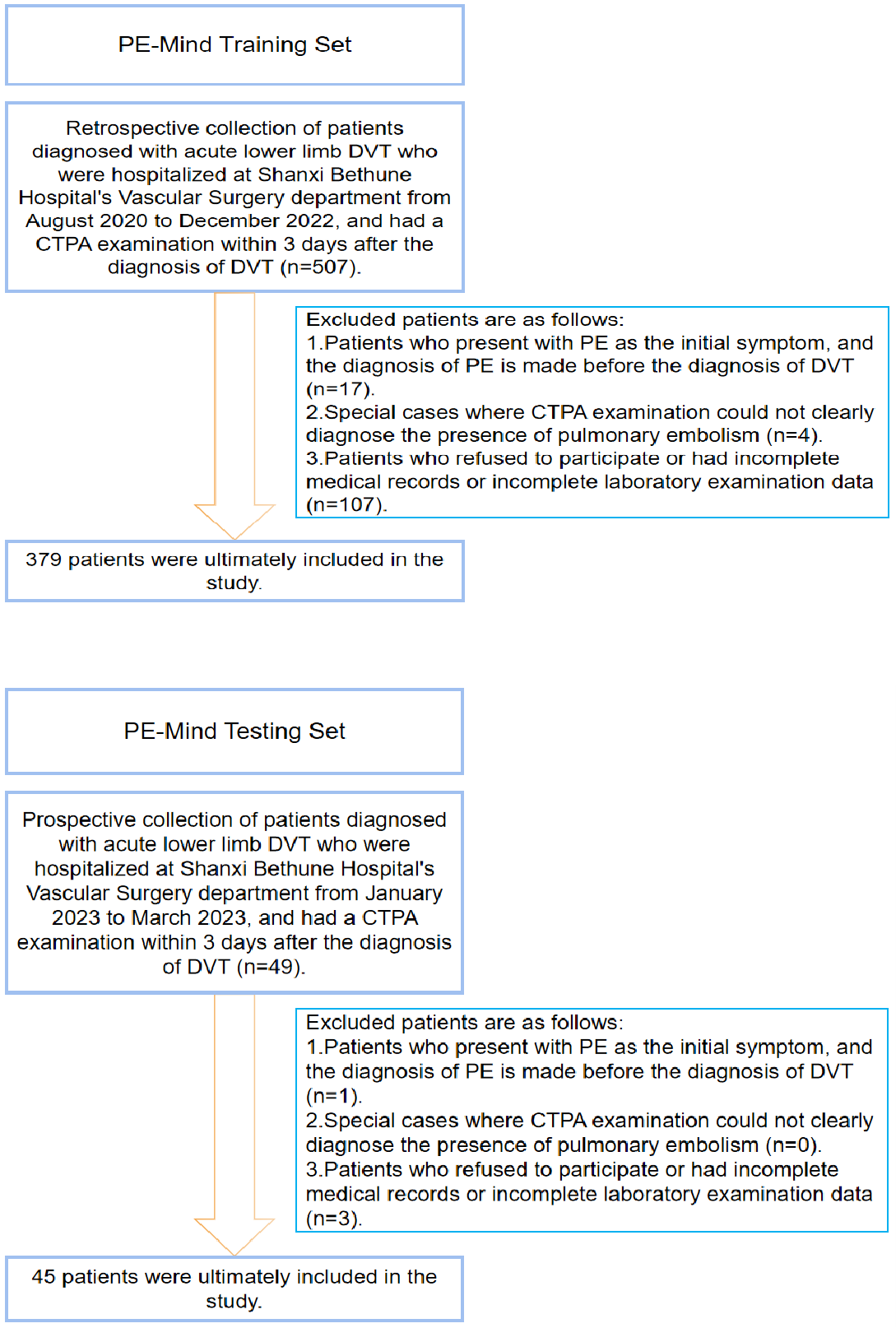

Supplement: SUPPLEMENTARY FIGURE 1 — The flowchart of the patient selection process. This figure shows the criteria and steps for including or excluding patients, ensuring a clear understanding of the study cohort’s composition. [file Image_1.PNG]

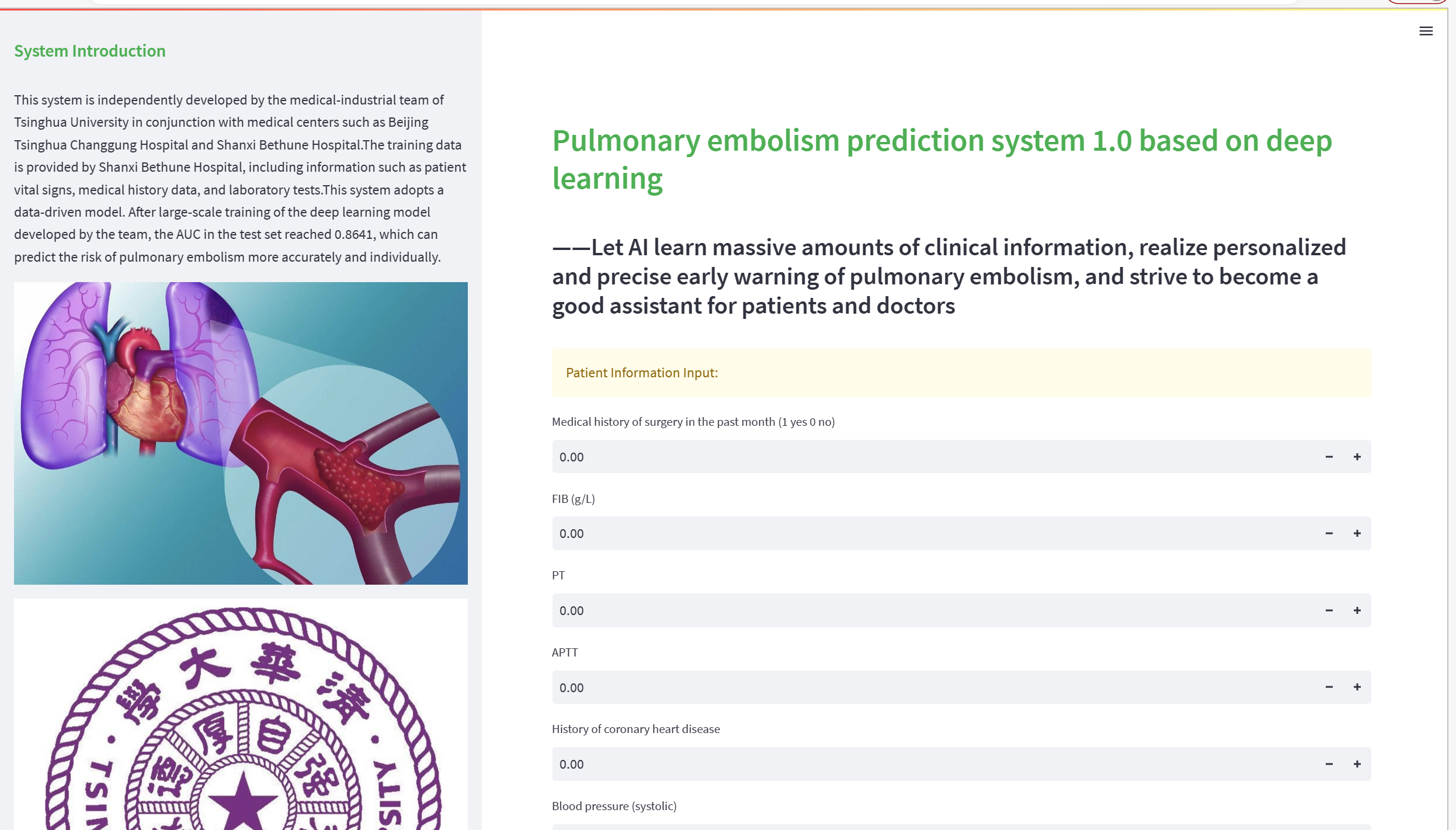

Supplement: SUPPLEMENTARY FIGURE 2 — Display of the operation interface of PulmoRiskAI. This figure provides a snapshot of the software interface, showcasing its user-friendly design and core functionalities for clinical application. [file Image_2.PNG]
