# Supplementary material for: Development and validation of a deep learning-enhanced prediction model for the likelihood of pulmonary embolism
Source: Front Med (Lausanne). 2025 Feb 6;12:1506363. doi: 10.3389/fmed.2025.1506363 (PMC11839595; doi:10.3389/fmed.2025.1506363)
Supplement: Supplementary file 3 [file Table_1.DOCX]

Table S1 Statistical results of clinical baseline data collected from patients.

| Characteristics | Non-PE | PE | P value |
| --- | --- | --- | --- |
| n | 255 | 169 |  |
| Sex 0=Female, 1=Male, n (%) |  |  | < 0.001 |
| 0 | 158 (37.3%) | 65 (15.3%) |  |
| 1 | 97 (22.9%) | 104 (24.5%) |  |
| Age (years), median (IQR) | 60 (52, 69) | 60 (49, 67) | 0.142 |
| Heart rate (beats/min), median (IQR) | 80 (72, 89) | 80 (76, 91) | 0.118 |
| Systolic blood pressure (mmHg), mean ± sd | 131.09 ± 16.233 | 133.15 ± 16.059 | 0.200 |
| Diastolic blood pressure (mmHg), mean ± sd | 78.157 ± 11.406 | 79.68 ± 10.68 | 0.168 |
| Respiratory rate (breaths/min), median (IQR) | 20 (19, 20) | 20 (19, 20) | 0.933 |
| Body temperature (°C), median (IQR) | 36.5 (36.2, 36.5) | 36.5 (36.3, 36.5) | 0.793 |
| Fingertip oxygen saturation, median (IQR) | 96 (95, 97) | 96 (95, 97) | 0.106 |
| Hypertension history 0=no, 1=yes, same as below, n (%) |  |  | 0.580 |
| 0 | 170 (40.1%) | 117 (27.6%) |  |
| 1 | 85 (20%) | 52 (12.3%) |  |
| Diabetes history , n (%) |  |  | 0.263 |
| 0 | 228 (53.8%) | 145 (34.2%) |  |
| 1 | 27 (6.4%) | 24 (5.7%) |  |
| Chronic heart failure history, n (%) |  |  | 1.000 |
| 0 | 253 (59.7%) | 167 (39.4%) |  |
| 1 | 2 (0.5%) | 2 (0.5%) |  |
| Chronic lung disease history, n (%) |  |  | 0.218 |
| 0 | 248 (58.5%) | 168 (39.6%) |  |
| 1 | 7 (1.7%) | 1 (0.2%) |  |
| Arterial embolism history, n (%) |  |  | 0.524 |
| 0 | 221 (52.1%) | 150 (35.4%) |  |
| 1 | 34 (8%) | 19 (4.5%) |  |
| Coronary artery disease history, n (%) |  |  | 0.451 |
| 0 | 242 (57.1%) | 163 (38.4%) |  |
| 1 | 13 (3.1%) | 6 (1.4%) |  |
| Cerebral infarction history, n (%) |  |  | 0.524 |
| 0 | 221 (52.1%) | 150 (35.4%) |  |
| 1 | 34 (8%) | 19 (4.5%) |  |
| Arterial stenosis history, n (%) |  |  | 0.705 |
| 0 | 235 (55.4%) | 154 (36.3%) |  |
| 1 | 20 (4.7%) | 15 (3.5%) |  |
| Atherosclerosis history, n (%) |  |  | 0.726 |
| 0 | 197 (46.5%) | 133 (31.4%) |  |
| 1 | 58 (13.7%) | 36 (8.5%) |  |
| Previous history of venous thrombosis 1=History of lower limb venous thrombosis 2=Pulmonary embolism 3=Two or more 4=None, n (%) |  |  | 0.744 |
| 1 | 10 (2.4%) | 4 (0.9%) |  |
| 2 | 1 (0.2%) | 1 (0.2%) |  |
| 3 | 3 (0.7%) | 1 (0.2%) |  |
| 4 | 241 (56.8%) | 163 (38.4%) |  |
| Presence of etiological factors, n (%) |  |  | 0.319 |
| 0 | 86 (20.3%) | 65 (15.3%) |  |
| 1 | 169 (39.9%) | 104 (24.5%) |  |
| Surgery History，n (%) |  |  | 0.848 |
| 0 | 214（50.5%） | 143（33.7%） |  |
| 1 | 41（9.7%） | 26（6.1%） |  |
| Immobilization history in the past month , n (%) |  |  | 0.676 |
| 0 | 202 (47.6%) | 131 (30.9%) |  |
| 1 | 53 (12.5%) | 38 (9%) |  |
| Cancer history, n (%) |  |  | 0.168 |
| 0 | 209 (49.3%) | 147 (34.7%) |  |
| 1 | 46 (10.8%) | 22 (5.2%) |  |
| Unilateral lower limb pain , n (%) |  |  | 0.466 |
| 0 | 101 (23.8%) | 61 (14.4%) |  |
| 1 | 154 (36.3%) | 108 (25.5%) |  |
| Unilateral swelling , n (%) |  |  | 0.513 |
| 0 | 39 (9.2%) | 22 (5.2%) |  |
| 1 | 216 (50.9%) | 147 (34.7%) |  |
| DVT occurring limb 1=left side 2=right side 3=bilateral, n (%) |  |  | 0.076 |
| 1 | 143 (33.7%) | 78 (18.4%) |  |
| 2 | 62 (14.6%) | 57 (13.4%) |  |
| 3 | 50 (11.8%) | 34 (8%) |  |
| Swelling of the limb on the side where DVT occurred , n (%) |  |  | 0.487 |
| 0 | 41 (9.7%) | 23 (5.4%) |  |
| 1 | 214 (50.5%) | 146 (34.4%) |  |
| Pain in the limb on which DVT occurs, n (%) |  |  | 0.682 |
| 0 | 94 (22.2%) | 59 (13.9%) |  |
| 1 | 161 (38%) | 110 (25.9%) |  |
| DVT site (1=femoral and iliac veins 2=popliteal veins and distal 3=full), n (%) |  |  | 0.093 |
| 1 | 38 (9%) | 22 (5.2%) |  |
| 2 | 132 (31.1%) | 73 (17.2%) |  |
| 3 | 85 (20%) | 74 (17.5%) |  |
| VTE family history, n (%) |  |  | 0.339 |
| 0 | 252 (59.4%) | 164 (38.7%) |  |
| 1 | 3 (0.7%) | 5 (1.2%) |  |
| White blood cell count (×10 µ), median (IQR) | 6.3 (4.9, 7.8) | 6.4 (5.2, 8.5) | 0.187 |
| Neutrophil count (×10 µ), median (IQR) | 4.03 (2.89, 5.41) | 4.31 (3.21, 6.2) | 0.074 |
| Lymphocyte count (×10 µ), median (IQR) | 1.49 (1.16, 1.825) | 1.4 (1.09, 1.8) | 0.206 |
| Eosinophils count (×10 µ), median (IQR) | 0.1 (0.06, 0.175) | 0.1 (0.04, 0.16) | 0.789 |
| Basophils count(×10 µ), median (IQR) | 0.03 (0.02, 0.04) | 0.03 (0.02, 0.04) | 0.541 |
| Haemoglobin (g/L), median (IQR) | 126 (111.5, 138.5) | 132 (114, 144) | 0.029 |
| Platelet count (×10 µ), median (IQR) | 217 (170.5, 264.5) | 206 (162, 255) | 0.202 |
| D-dimer (ng/ml), median (IQR) | 637 (306.5, 2025.5) | 1270 (658, 2796) | < 0.001 |
| PT(s), median (IQR) | 11.8 (11.2, 12.55) | 12.2 (11.5, 13.1) | < 0.001 |
| APTT(s), median (IQR) | 31.7 (29.2, 34.8) | 31.7 (29.3, 34.3) | 0.533 |
| TT(s), median (IQR) | 14 (13.3, 15) | 13.7 (13.1, 15) | 0.390 |
| FIB (g/L), median (IQR) | 3.31 (2.73, 3.91) | 3.32 (2.84, 4.01) | 0.403 |
| Antithrombin III activity (%), median (IQR) | 99 (90, 108) | 95 (87, 104) | 0.020 |
| ALT (U/L), median (IQR) | 17.2 (11.95, 27.2) | 18.1 (11.8, 27.9) | 0.748 |
| AST (U/L), median (IQR) | 19.5 (15.8, 26.95) | 19.1 (15.4, 27.5) | 0.601 |
| Serum albumin (g/L), median (IQR) | 37.4 (33.75, 40) | 36.9 (33.8, 39.5) | 0.545 |
| Blood creatinine (umol/L), median (IQR) | 70.6 (62.65, 79.65) | 75.3 (65.9, 85.3) | 0.001 |
| Serum potassium (mmol/L), mean ± sd | 3.8624 ± 0.35684 | 3.9307 ± 0.36406 | 0.056 |
| Serum sodium (mmol/L), median (IQR) | 138.9 (136.9, 140.4) | 138.7 (136.5, 140.2) | 0.255 |
| Serum chloride (mmol/L), median (IQR) | 105.2 (102.85, 107.15) | 104.7 (102.3, 106.4) | 0.047 |

Note: DVT: Deep Vein Thrombosis; PT: Prothrombin Time; APTT: Activated Partial Thromboplastin Time; TT: Thrombin Time; FIB: Fibrinogen; ALT: Alanine Aminotransferase; AST: Aspartate Aminotransferase. ALT: Alanine Aminotransferase; AST: Aspartate Aminotransferase.

Table S2 Principal Component Analysis (PCA) Contributions of Top 37 Clinical Factors for Pulmonary Embolism Risk Assessment

| Factor | PCA Contribution |
| --- | --- |
| Surgery History | 0.410171 |
| Fibrinogen | 0.358052 |
| Prothrombin Time | 0.348968 |
| Activated Partial Thromboplastin Time | 0.310127 |
| Coronary Heart Disease History | 0.240626 |
| Hypertension History | 0.217242 |
| VTE Family History | 0.189477 |
| D-Dimer | 0.187352 |
| Arterial Stenosis History | 0.177364 |
| Antithrombin III Activity | 0.170385 |
| Basophilic Count | 0.152981 |
| Arteriosclerosis History | 0.141174 |
| Body Temperature | 0.137686 |
| Presence of Etiological Factors | 0.136228 |
| Sex | 0.132743 |
| Platelet Count | 0.131469 |
| Thrombin Time | 0.125334 |
| Immobilization History | 0.123982 |
| High Blood Pressure History | 0.123497 |
| Venous Thrombosis History | 0.109457 |
| Blood Oxygen Saturation | 0.10425 |
| Age | 0.090734 |
| Cancer History | 0.088844 |
| Respiratory Rate | 0.081267 |
| Aspartate Aminotransferase | 0.074987 |
| Chronic Lung Disease History | 0.074498 |
| Blood Potassium | 0.073202 |
| Alanine Aminotransferase | 0.072602 |
| Heart Rate | 0.066132 |
| Chronic Heart Failure History | 0.052186 |
| Unilateral Lower Limb Swelling | 0.051072 |
| DVT Location | 0.044974 |
| Diabetes History | 0.036445 |
| Unilateral Lower Limb Pain | 0.030719 |
| Cerebral Infarction History | 0.029569 |
| Arterial Embolism History | 0.029569 |
| DVT Affected Limb | 0.025975 |
